# Reconfigurable Self‐Assembling Photocatalytic Magnetic Liquid Metal Microrobot Swarm for Microplastic Capture and Degradation

**DOI:** 10.1002/smll.202501351

**Published:** 2025-08-27

**Authors:** Xianghua Wu, Xia Peng, Long Ren, Jianguo Guan, Martin Pumera

**Affiliations:** ^1^ Future Energy and Innovation Laboratory Central European Institute of Technology Brno University of Technology Purkynova 123 Brno 61200 Czech Republic; ^2^ State Key Laboratory of Advanced Technology for Materials Synthesis and Processing International School of Materials Science and Engineering Wuhan University of Technology 122 Luoshi Road Wuhan 430070 China; ^3^ Wuhan Institute of Photochemistry and Technology 7 North Bingang Road Wuhan 430083 China; ^4^ Department of Medical Research China Medical University Hospital China Medical University No. 91 Hsueh‐Shih Road Taichung TW‐40402 Taiwan; ^5^ Advanced Nanorobots & Multiscale Robotics Laboratory Faculty of Electrical Engineering and Computer Science VSB ‐Technical University of Ostrava 17. listopadu 2172/15 Ostrava 70800 Czech Republic

**Keywords:** liquid metal, microplastics, robotics, swarm

## Abstract

Microplastic pollution has emerged as a global environmental concern, requiring effective methods for its capture and removal from ecosystems. Inspired by natural swarming behaviors, micro/nanorobot swarms are developed to address challenges in fields such as environmental remediation. An innovative solution is presented designing reconfigurable and regenerable liquid metal microrobots (LiquidBots) made from bio‐friendly gallium‐based liquid metal. These LiquidBots can self‐assemble into swarms and actively capture microplastics through electrostatic interactions. They can be regenerated via ultrasonic treatment, allowing for repeated use without loss of efficiency. This approach offers an efficient, sustainable, and adaptable solution to the growing problem of microplastic pollution in aquatic environments.

## Graphical abstract

Inspired by natural swarming behaviors, reconfigurable and regenerable gallium‐based liquid metal microrobots (LiquidBots) are developed for efficient microplastic removal. These microrobots self‐assemble into swarms, capture microplastics via electrostatic interactions, and can be regenerated through ultrasonic treatment. This sustainable, reusable strategy offers a promising solution for addressing microplastic pollution in aquatic environments.

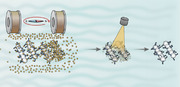

## 1 Introduction

The phenomenon of swarming is widespread in various biological systems in nature, demonstrating the ability of organisms to accomplish complex tasks through swarming behavior that individuals alone cannot achieve.^[^ ^1^, ^2^, ^3^ ^]^ Group cooperation usually refers to the coordination of a group of individuals working together as a unified collective to perform activities such as foraging. This behavior is particularly evident in insects and mammals. For example, ant and bee colonies exhibit highly organized social structures, efficiently accomplishing tasks like foraging through clear division of labor and cooperation.^[^ ^4^, ^5^ ^]^ Mammals such as wolves and lions increase their hunting success rate significantly through cooperative hunting.^[^ ^6^, ^7^ ^]^ Swarming behavior in nature not only showcases the communication and coordination abilities between individuals but also highlights the powerful advantages of collective intelligence.^[^ ^8^ ^]^ Inspired by this natural phenomenon, researchers have developed micro/nanorobot swarms to tackle various complex tasks.^[^ ^9^, ^10^, ^11^ ^]^

Micro/nanorobot swarms are intelligent devices capable of operating at the micron and nanoscale, typically made from advanced materials driven by magnetic fields, light, ultrasound fields, electric fields, or chemical means.^[^ ^12^, ^13^, ^14^, ^15^, ^16^, ^17^, ^18^, ^19^ ^]^ Due to their small size and highly controllable structures, micro/nanorobots (MNRs) play an important role in fields such as environmental remediation, drug delivery, and medical diagnostics.^[^ ^20^, ^21^, ^22^, ^23^, ^24^, ^25^, ^26^, ^27^ ^]^ For instance, in the biomedical field, micro/nanorobot swarms can enter the human body, precisely target diseased areas, and perform targeted treatments.^[^ ^28^, ^29^ ^]^ In environmental remediation, microplastic pollution has become a pressing global issue, with these persistent pollutants found in oceans, freshwater bodies, and even the air. Notable innovations include the use of microrobot swarms, which can dynamically capture free‐floating bacteria and dispersed microplastics, aiding in water purification.^[^ ^30^ ^]^ In particular, self‐propelled, light‐controlled hematite microrobots have shown great potential, as they can navigate aqueous environments and break down polymer chains through photocatalytic and Fenton‐like reactions.^[^ ^31^ ^]^ However, despite significant progress in this field, current MNRs used for environmental applications primarily rely on ultraviolet (UV) light for degradation, and most existing MNRs are single‐use, lacking the ability to be regenerated.^[^ ^24^, ^25^, ^26^, ^32^ ^]^ Even those designed for reuse experience a sharp decline in efficiency after several uses, ultimately becoming non‐functional.^[^ ^27^, ^33^ ^]^ Therefore, developing MNRs swarm with reconfigurable and regenerable capabilities that can capture and degrade micro/nanoplastics under natural light remains an important area for future exploration.

Herein, we introduce a dual‐field‐driven liquid metal microrobots (LiquidBots) swarm with reconfigurable and regenerable capabilities. This system, driven by both magnetic and light fields, can actively capture and degrade microplastics in water, offering an innovative solution to environmental remediation. Bio‐friendly gallium‐based liquid metal was employed as the foundation and subsequently coated with WO_x_ on the surface through an advanced interface engineering strategy to successfully construct LiquidBots with reconfigurable and regenerable properties.^[^ ^34^, ^35^, ^36^ ^]^ These LiquidBots not only exhibit excellent performance but also maintain stable self‐propulsion capabilities in complex water environments. LiquidBots exhibit self‐propulsion behavior under both natural sunlight and UV light (365 nm), attributed to the photocatalytic activity of the WOx coating. They are capable of self‐assembly into long chains, ascribed to the magnetic interactions of the ferromagnetic iron nanoparticles. Under the precise control of a 3D magnetic field, these chains can move directionally and efficiently to capture microplastics dispersed in the water through electrostatic interactions. To verify their degradation efficiency, matrix‐assisted laser desorption/ionization mass spectrometry (MALDI‐MS) was used to determine the degradation of high‐molecular weight (*M*w 4000) polyethylene glycol (PEG) by LiquidBots. This is because PEG can be quickly and conveniently detected and analyzed using MALDI‐MS. More importantly, after completing their tasks, LiquidBots can be regenerated through successive ultrasonic treatments in different solutions, making them reusable. This regenerable property greatly enhances their economic viability and sustainability, overcoming the limitations of traditional single‐use MNRs. Through this innovative design, we provide an intelligent and sustainable solution for environmental remediation, with the potential for profound impacts in water purification and microplastics pollution degradation in the future.

## 2 Results and Discussion

**Figure 1:**
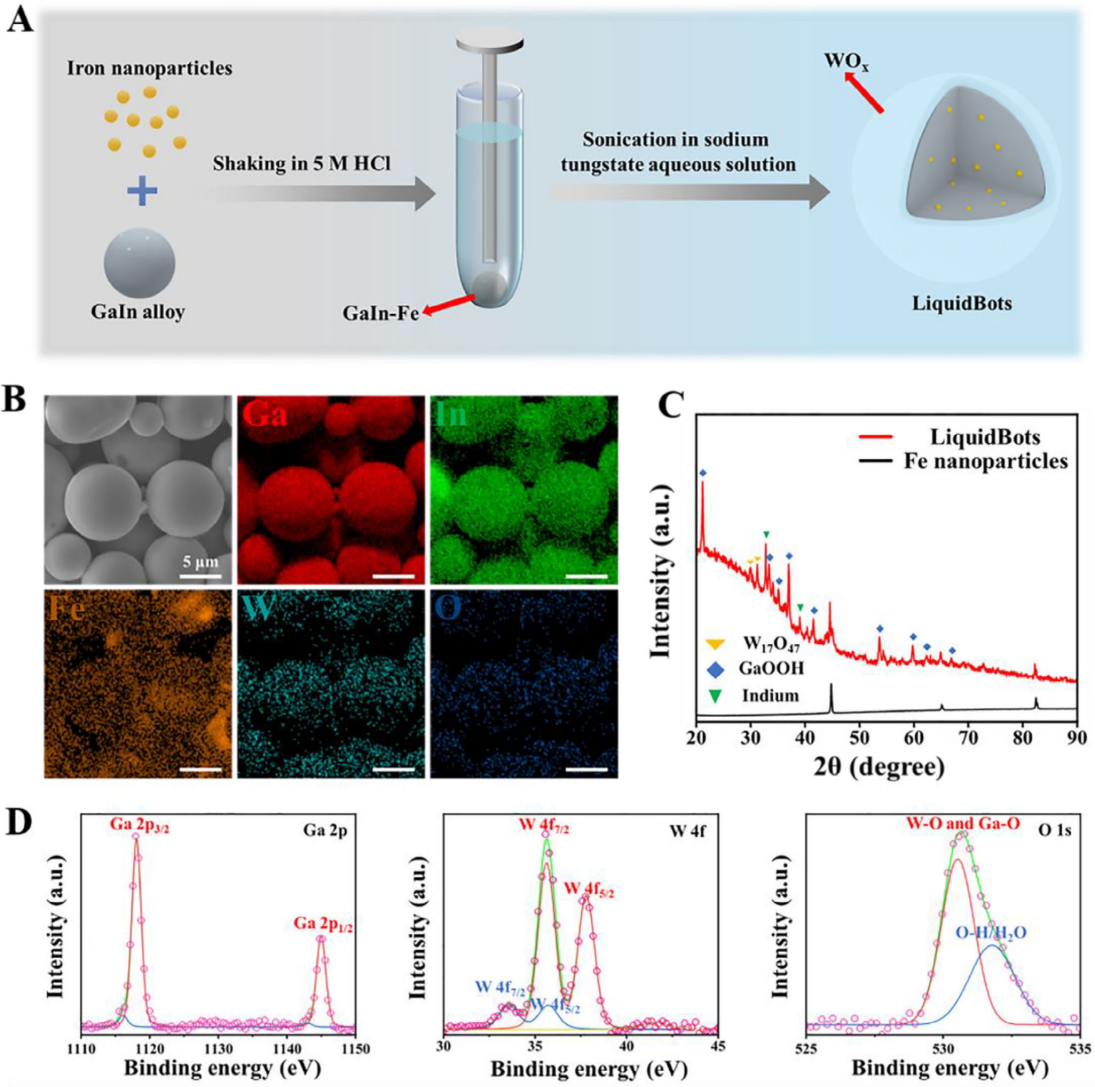
Synthesis and characterization of LiquidBots. A) Schematic representation of the synthesis process. Reproduced (Adapted) under the terms of the Creative Commons CC‐BY license.^[^ ^38^ ^]^ Copyright 2024, Wiley‐VCH. B) SEM images with corresponding EDX mapping of LiquidBots. Scale bar: 5 µm. C) XRD patterns of LiquidBots and Fe nanoparticles. D) Ga 2p, W 4f, and O 1s spectra of LiquidBots.

As an initial step toward LiquidBots (as illustrated in **Figure** **1**A), a mixture of iron (Fe) nanoparticles within the liquid metal (GaIn‐Fe) matrix was prepared according to previous work.^[^ ^37^, ^38^ ^]^ Because the reducing potential of WO_4_^2−^ is higher than that of Ga/Ga^3+^, a galvanic reaction was induced on the newly formed fresh metallic Ga surface when GaIn‐Fe was sonicated in tungstate (WO_4_^2−^).^[^ ^39^, ^40^ ^]^ To reveal possible changes in the morphology and elemental composition of the obtained particles, scanning electron microscopy (SEM) and energy dispersive X‐ray spectroscopy (EDX) were used (Figure S1, Supporting Information). As shown in Figure 1B, the LiquidBots exhibit a smooth surface and clear core–shell spherical structure. From the EDX results, signals for Ga, In, and Fe were detected in the core while signals for tungsten (W) and oxygen (O) were observed throughout the LiquidBots, indicating a core–shell structure and proving the presence of WO_x_ layers on the surface. The X‐ray diffraction (XRD) pattern of Fe nanoparticles revealed three distinct peaks at 44.8°, 64.1°, and 82.4° (JCPDF 06–0696), corresponding to the (220), (321), and (332) crystal planes, respectively (Figure 1C). These same peaks were also observed in the XRD patterns of the LiquidBots sample, confirming the successful integration of LiquidBots. In addition, the XRD patterns of W_17_O_47_ revealed two peaks at 29.7° and 31.1° (JCPDF 44–0396), corresponding to the (004) and (104) crystal planes, respectively. X‐ray photoelectron spectroscopy (XPS) was performed to analyze the surface composition of LiquidBots (Figure S2, Supporting Information; Figure 1D). In the high‐resolution spectra, Ga 2p_1/2_ (1145 eV) and Ga 2p_3/2_ (1118 eV) peaks were observed, with a separation of 26.9 eV, consistent with the binding energies of Ga 2p, confirming the presence of gallium oxide (Ga^3+^). Peaks at 1143 and 1116 eV correspond to the metallic state of Ga. The strong peak at 530.5 eV is associated with W─O and Ga─O bonding while the peak at 531.8 eV is attributed to surface hydroxyl groups. Peaks at 33.6 and 35.7 eV correspond to WO_2_, and peaks at 35.7 and 37.8 eV correspond to WO_3_.^[^ ^39^, ^40^, ^41^ ^]^ The above results indicate that the composite material has a core–shell structure, further confirming the presence of a WO_x_ layer on its surface.

**Figure 2:**
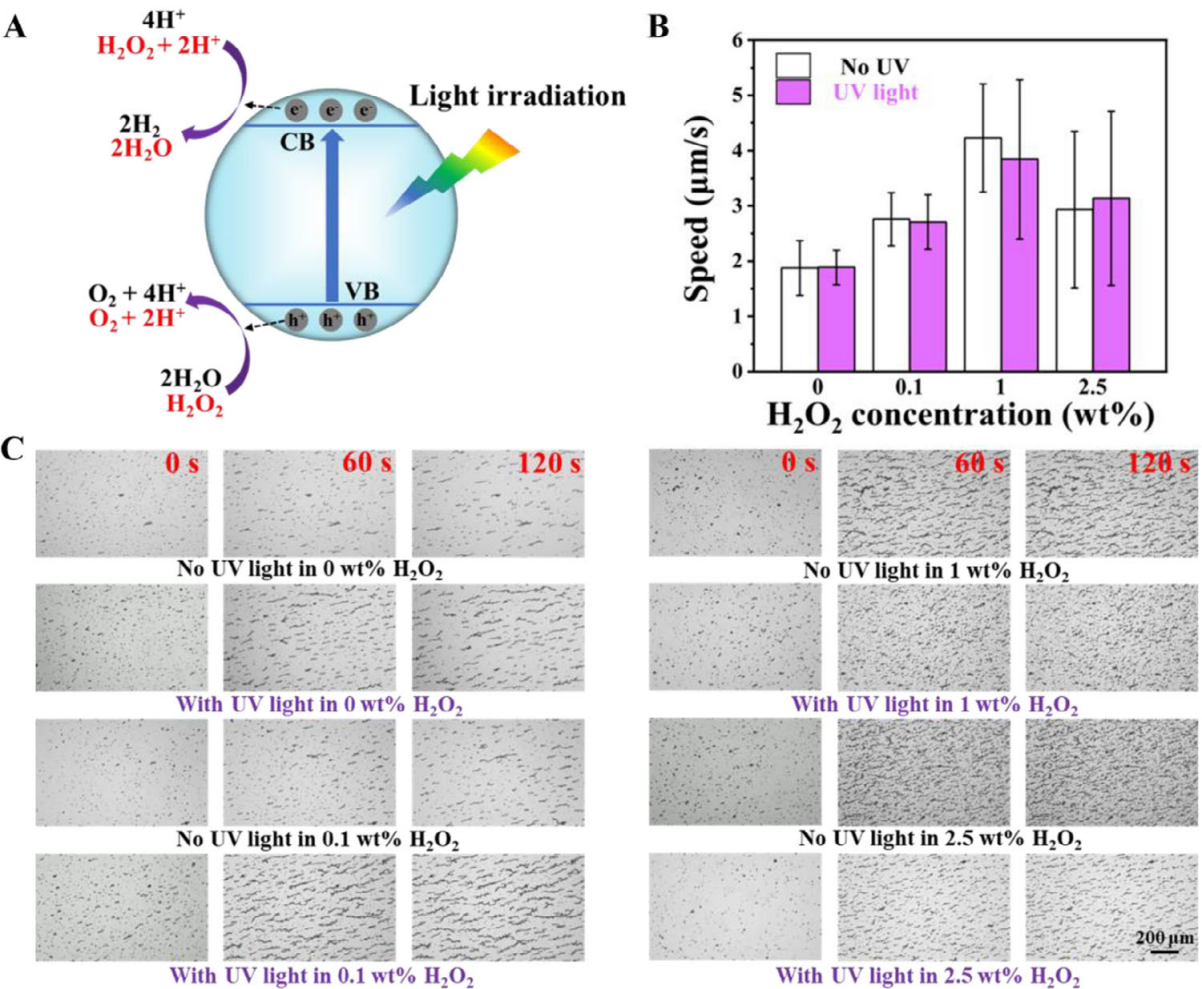
Propulsion mechanism, speed, and swarming behavior of LiquidBots under different conditions. A) Schematic illustration of the propulsion mechanism of light‐driven LiquidBots. B) Speed at different concentrations of H_2_O_2_ with/without UV light. C) Self‐assembly of LiquidBots in different concentrations of H_2_O_2_ with/without UV‐light irradiation. Scale bar: 200 µm.

The LiquidBots exhibit autonomous propulsion in water with minimal hydrogen peroxide (H_2_O_2_) fuel. This ability, activated under natural or UV irradiation, is essential for their potential applications, including self‐assembly swarms for capturing amino‐modified polystyrene (PS) microplastics with diameters of 2 µm and degrading PEG. Meanwhile, WO_x_, a semiconducting material, shows photocatalytic properties when exposed to a light source.^[^ ^42^ ^]^ The general motion mechanism of light‐propelled LiquidBots is shown in **Figure** **2**A. Figure 2B illustrates the comparison of the LiquidBots’ speed under various conditions. LiquidBots show similar speed under irradiation by either white or UV light. As the concentration of H_2_O_2_ increased, the LiquidBots gradually accelerated. When the H_2_O_2_ concentration reached 1 wt.%, the LiquidBots achieved their peak speed of ≈4 µm s^−1^. This indicates that at optimal concentrations, H_2_O_2_ can significantly enhance the self‐propelling capability of LiquidBots, allowing them to move and function more effectively in water. However, as the H_2_O_2_ concentration was further increased, the speed of the LiquidBots decreased. This phenomenon is likely due to increased ionic strength in the system due to excessive H_2_O_2_ concentration, which inhibits the LiquidBots’ self‐propulsion ability.^[^ ^43^ ^]^ To further investigate the behavior of LiquidBots under different concentrations of H_2_O_2_ and in water environments, we studied their aggregation and chain formation as shown in Figure 2C. Initially, the LiquidBots were randomly distributed in solution; over time, they began to self‐assemble into long chains due to the ferromagnetic properties of magnetic Fe nanoparticles within the LiquidBots (Figure S3, Supporting Information). During the self‐propulsion process, magnetic interactions caused the LiquidBots to move closer together and aggregate into chain‐like structures. As the experiment progressed, the LiquidBots formed increasingly longer chains, with the longest chains observed at an H_2_O_2_ concentration of 1 wt.%. This phenomenon is attributed to the peak self‐propulsion speed at this concentration, which enhances collision frequency and accelerates chain formation. We compared the time required for LiquidBots to form chains of ≈25 µm in 0 and 1 wt.% H_2_O_2_ (Figure S4, Supporting Information). The results indicate that in 0 wt.% H_2_O_2_, LiquidBots took 48 s, whereas in 1 wt.% H_2_O_2_, they required only 8 s to reach the same length. This confirms that higher self‐propulsion speed significantly accelerates chain formation. These rapidly formed long‐chain structures not only provide greater coverage but also maintain high efficiency in capturing particles in dynamic environments.

**Figure 3:**
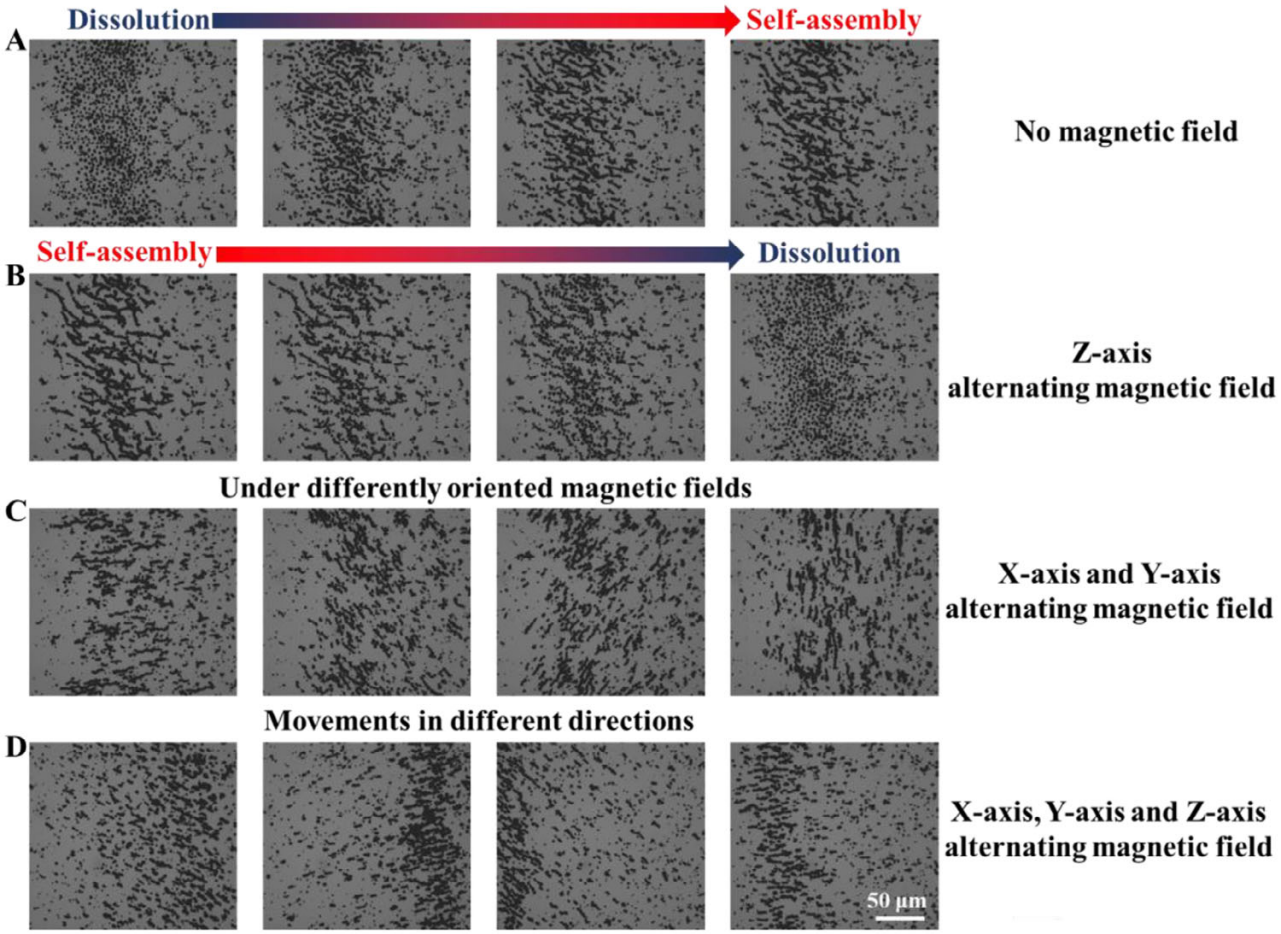
States of LiquidBots swarms under different conditions. A) No magnetic field. B) Z‐axis alternating magnetic field. C) X‐axis and Y‐axis alternating magnetic field. D) X‐axis, Y‐axis, and Z‐axis alternating magnetic field. Scale bar: 50 µm.

Highly programmable manipulation of LiquidBots was achieved through precise control of a 3D magnetic field. In the absence of an external magnetic field, LiquidBots spontaneously aggregated into long chains, typically aligning with the geomagnetic field (**Figure** **3**A). This phenomenon is due to the magnetic interactions between the ferromagnetic nanoparticles embedded in the LiquidBots. However, when an alternating magnetic field was applied along the Z‐axis, the long chains of LiquidBots began to disassemble, becoming shorter and eventually dispersing uniformly in the solution (Figure 3B). When an alternating magnetic field was applied along the X‐ or Y‐axis, the LiquidBots chains realigned to match the direction of the external magnetic field (Figure 3C). Furthermore, by applying alternating magnetic fields along the X, Y, and Z axes simultaneously, the long chains of LiquidBots moved in different spatial directions in response to the changes in the 3D magnetic field (Figure 3D). This multi‐dimensional magnetic control greatly enhances the functionality of LiquidBots, enabling precise navigation and dynamic adjustments in complex environments (Movie S1, Supporting Information).

**Figure 4:**
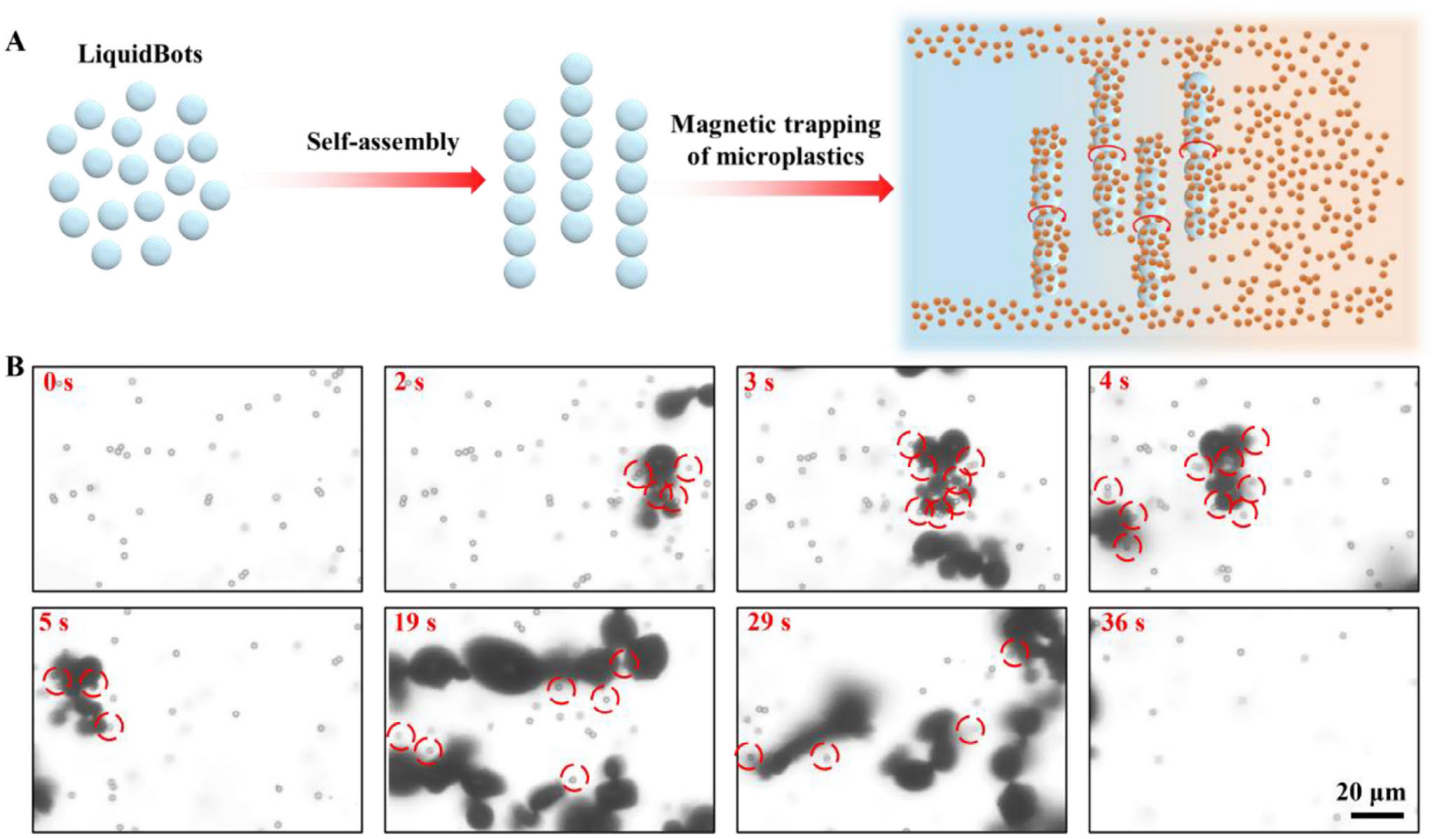
LiquidBots swarm to capture microplastics. A) Schematic illustration of LiquidBots capturing microplastics under an external magnetic field after self‐assembly. B) Time‐lapse images of LiquidBots removing microplastics. Scale bar: 20 µm.

The long‐chain structure of LiquidBots offers an ideal solution for removing micro/nanoplastics from water (**Figure** **4**A). It is highlighted that LiquidBots can self‐assemble into long chains, significantly increasing their coverage and thus improving the efficiency of microplastics removal. Additionally, the high flexibility of LiquidBots allows them to adapt to complex water environments, demonstrating immense potential in practical applications. As shown in Figure S5 (Supporting Information), we first evaluated the movement speed of LiquidBots at different magnetic field frequencies by adjusting the magnetic field parameters. When the magnetic field strength was set to 3 mT, the speed of the LiquidBots increased with the magnetic field frequency for frequencies below 30 Hz. However, within the 30–50 Hz range, the LiquidBots’ speed temporarily decreased before rising again as the frequency increased. This phenomenon is related to the unique liquid–solid configuration of LiquidBots, where the Fe nanoparticles inside the liquid metal respond to external magnetic fields and undergo restructuring. When the frequency exceeds 70 Hz, the LiquidBots’ speed declines sharply as the magnetic torque becomes insufficient to maintain synchronization between the magnetic moment and the external magnetic field.^[^ ^13^ ^]^ The time‐lapse images in Figure 4B show the gradual formation of long chains by LiquidBots under the influence of a magnetic field and how these chains move to capture microplastics in the water (Movie S2, Supporting Information). Initially, the microplastics were uniformly dispersed in the water. As the LiquidBots were introduced, their self‐propelling properties caused them to assemble into long chains. When a rotating magnetic field is applied, the negatively charged LiquidBots' long‐chain structures move under magnetic actuation and approach the microplastics in the solution. Through electrostatic interactions, they preferentially adsorb positively charged microplastics. After the initial adsorption, the gallium oxide layer on the LiquidBots' surface further enhances adhesion, firmly securing the microplastics in place (Figure S6, Supporting Information).^[^ ^44^ ^]^ By utilizing the proposed mechanism, the LiquidBots successfully removed ≈80% of the microplastics from the water within 36 s, showcasing their exceptional pollution treatment capabilities. This efficient and flexible LiquidBots platform provides a promising solution for removing microplastics from water.

**Figure 5:**
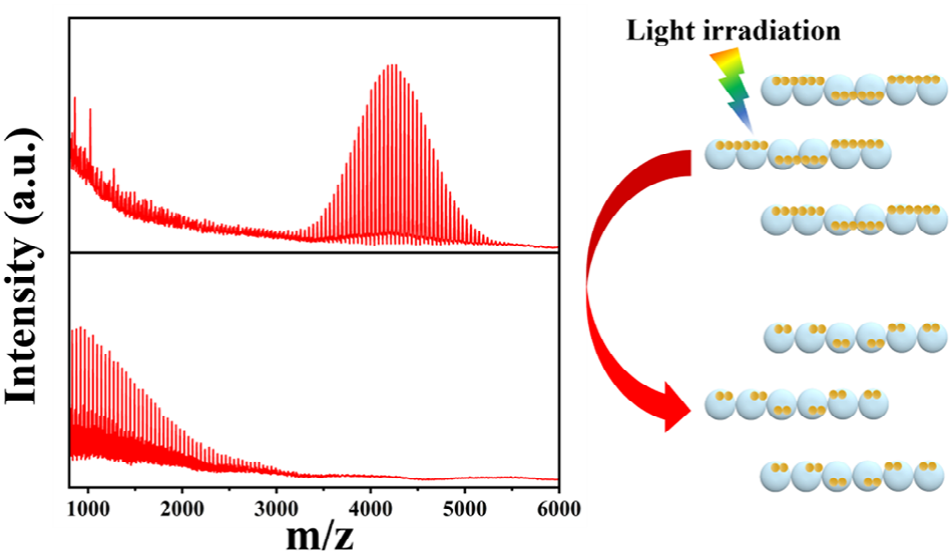
PEG chain photodegradation. From top to bottom: MALDI‐MS spectra of untreated PEG and PEG treated with LiquidBots in 1 wt.% H_2_O_2_ for 6 h.

PEG‐4000 was selected instead of polystyrene primarily because of its well‐defined molecular weight and the ease with which its degradation fragments can be clearly characterized using MALDI‐MS, thereby facilitating analysis of the degradation process. First, the Zeta potential of PEG 4000 was tested, revealing that PEG 4000 carries a positive charge in water, whereas LiquidBots carry a negative charge (Figure S6, Supporting Information). Thus, during the light‐driven formation of LMMR microchains, the electrostatic interactions crucial for improving polymer capture efficiency significantly enhance the LiquidBots’ ability to capture PEG. To further enhance degradation efficiency, the experiment employed a high concentration of H_2_O_2_ (1 wt.%) to increase the self‐propulsion speed and break the strong covalent bonds within the PEG polymer chains through enhanced photocatalytic activity. The synergistic effect of high‐concentration H_2_O_2_ and LiquidBots contributed to generate more reactive oxygen species (ROS), thereby accelerating PEG degradation.^[^ ^25^, ^26^, ^44^ ^]^ Under the action of the self‐assembled microchains cube‐shaped microrobots, PEG was exposed to natural light for 6 h. **Figure** **5** shows the MALDI‐MS spectra of untreated PEG and PEG solutions treated with microrobots in 1 wt.% H_2_O_2_. After 6 h of natural light exposure, the PEG solution treated with microrobots showed the complete disappearance of the signal corresponding to the molecular weight of the PEG macromolecule chain. The degradation of PEG microplastics is driven by a combination of photocatalysis and the photo‐Fenton reaction. Under visible light irradiation, WOx is excited to generate electron‐hole pairs:

(1) $$WO_{x}+h\nu \to e^{-}+h^{+}$$

The electrons (*e* ^−^) react with dissolved oxygen (O_2_) to generate superoxide radicals (O_2_^· −^):

(2) $$O_{2}+e^{-}\to {O_{2}}^{\cdot -}$$

The H_2_O_2_ can also combine with electrons, further generating hydroxyl radicals (· OH) and hydroxide ions (OH^−^):

(3) $$H_{2}O_{2}+e^{-}\to \cdot \mathrm{OH}+OH^{-}$$

The holes (h^+^) oxidize water or hydroxide ions to produce hydroxyl radicals (· OH):

(4) $$H_{2}O+h^{+}\to \cdot \mathrm{OH}+H^{+}$$

(5) $$OH^{-}+h^{+}\to \cdot \mathrm{OH}$$

These reactive oxygen species (ROS) attack the C─O bonds in the PEG main chain, ultimately breaking it down into smaller molecules or fragments.

Control experiments were conducted to further demonstrate the effectiveness of this chain structure in degrading PEG. In the absence of LiquidBots, where only 1 wt.% H_2_O_2_ was added, the MALDI‐MS spectrum of PEG showed almost no change in the signal for the PEG macromolecular chain molecular weight (near 4000). This indicates that H_2_O_2_ alone was insufficient to degrade PEG and highlights the critical role LiquidBots play in the process (Figure S7, Supporting Information). The unique design of LiquidBots allows them not only to capture microplastics but also to accelerate significantly the degradation process through photocatalysis and electrostatic interactions, thus demonstrating their great potential for water purification applications.

**Figure 6:**
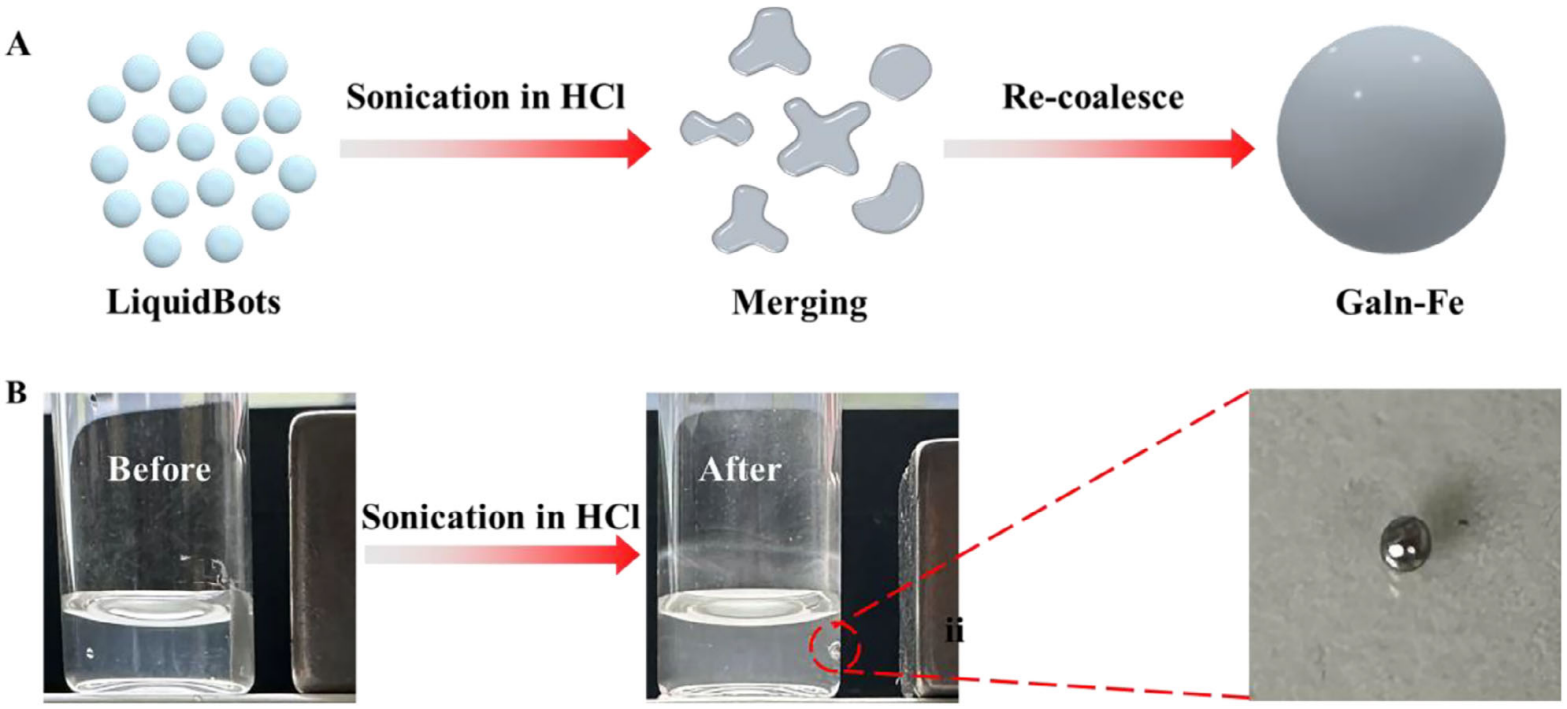
Regeneration process of GaIn‐Fe. A) Schematic diagram of the regeneration process of GaIn‐Fe. B) Optical image of LiquidBots before and after sonication in hydrochloric acid (HCl).

Additionally, after the removal of microplastic pollutants from water, LiquidBots demonstrate significant potential for recovery and reuse. This capability not only offers an economically efficient solution for pollution control but also significantly reduces resource waste and environmental burden. Sustainability makes LiquidBots particularly attractive for practical applications, especially in addressing global challenges like water scarcity and increasing pollution. Therefore, ensuring the safe regeneration and reuse of LiquidBots is crucial. Through well‐designed regeneration steps, the environmental impact can be minimized while also extending the lifespan of LiquidBots, allowing them to maintain high efficiency over multiple cycles. The LiquidBots’ regeneration process consists of three main steps to ensure complete functional recovery (as shown in **Figure** **6**A). The first step is to swiftly collect LiquidBots from the water using a magnet, a highly efficient and quick process that prevents the LiquidBots from remaining in the environment after pollutant removal. The second step involves placing the collected LiquidBots in an acid solution and using ultrasonic treatment for 2 min. During this process, the gallium oxide on the LiquidBots’ surface reacts with the acid solution to form soluble salts, exposing the liquid metal Ga. Figure 6B shows the state of the LiquidBots before and after ultrasonic treatment in an acid solution. The ultrasonic shear forces promote the LiquidBots to re‐coalesce into GaIn‐Fe alloy. To evaluate the reusability of the LiquidBots, their mass was recorded both before and after re‐coalescence into GaIn‐Fe. The re‐coalesced GaIn‐Fe showed only a 7.8% reduction in mass, confirming the LiquidBots' strong potential for repeated use with minimal material loss (Figure S8, Supporting Information). This ultrasound‐driven self‐repair mechanism offers the LiquidBots excellent structural stability and durability. In the third step, the LiquidBots are treated with sodium tungstate solution, allowing the entire structure to regenerate and return to its original state, ready for the next round of pollutant treatment. These results demonstrate that reconfigurable and regenerable LiquidBots hold immense potential for future environmental remediation applications due to their high recyclability and excellent reusability.

## 3 Conclusion

This study introduces dual‐field‐driven LiquidBots specifically designed for the targeted capture and degradation of microplastics in aquatic environments. LiquidBots were fabricated through the ultrasonic processing of a mixture of liquid metal and Fe nanoparticles in a sodium tungstate solution. These LiquidBots possess the capability to self‐assemble into long chains, employing electrostatic interactions to efficiently capture microplastics under the influence of an external magnetic field. Notably, LiquidBots achieved an 80% removal rate of microplastics within 36 s and facilitated the effective photodegradation of microplastics. Following the processing of microplastics, LiquidBots can be regenerated by ultrasonic treatment, reducing resource consumption and mitigating environmental risks, thus providing a sustainable solution for water purification. This approach aligns environmental protection with economic benefits, making it a key player in water resource management.

## 4 Experimental Section

### Fabrication of GaIn‐Fe and LiquidBots

Gallium (Merck, 99.999%) and indium (Merck, 99.999%) were mixed in a 3:1 ratio to form a GaIn alloy. This alloy, along with Fe nanoparticles (Aladdin, 100–300 nm, 99.9%), was added to 5 mol L^−1^ HCl (Merck, 37%) and shaken until the Fe fully infiltrated the GaIn alloy. The GaIn‐Fe alloy was extracted and then poured into a sodium tungstate dihydrate (Merck, ≥ 99%) solution (0.1 mmol L^−1^). After 10 min of ultrasonication at 100 W, the mixture was washed and filtered through a magnetic screen to obtain LiquidBots.

### Characterization of LiquidBots

A Tescan MIRA 3 XMU SEM with an Oxford EDX detector was used for sample morphology and elemental mapping. LiquidBots’ crystalline structure was analyzed using a Rigaku SmartLab 9 kW diffractometer with a Cu Kα X‐ray tube (45 kV, 200 mA). Magnetic hysteresis loops were measured at 300 K with a Quantum Design VersaLab Vibrating Sample Magnetometer (VSM), applying a magnetic field from −15 to 15 kOe. LiquidBots’ chemical composition was analyzed via XPS (Kratos Axis Supra) using an Al Kα source (1486.7 eV) and fitted with CasaXPS software. Zeta potential measurements for microplastics and LiquidBots in deionized water were taken with a Malvern Zetasizer Ultra, with triplicate runs to assess error.

### Light‐Driven Motion Experiments

The motion of LiquidBots under light activation was recorded using a Nikon ECLIPSE TS2R inverted microscope equipped with a Basler digital camera (acA1920‐155uc). In a typical procedure, LiquidBots were deposited onto a glass slide, followed by the addition of H_2_O_2_ (Merck, 30%) at concentrations of 0, 0.1, 1, and 2.5% to evaluate their behavior under different chemical conditions. All experiments were conducted without surfactants. The LiquidBots were then activated using either natural light or a 365 nm UV LED source (CoolLED pE‐100), with the UV light intensity measured at 1.6 W cm^−^^2^. Videos were recorded at 25 frames per second and analyzed using NIS Elements Advanced Research software to extract trajectories and calculate velocities.

### Magnetic‐Driven Motion Experiments

The motion of LiquidBots was recorded using a Nikon ECLIPSE TS2R microscope and a BASLER acA1920‐155uc camera, without surfactants. A custom magnetic setup with three orthogonal coil pairs on a PLA support generated a transverse rotating magnetic field. The microrobots were controlled under 3 mT magnetic fields at frequencies from 0 to 100 Hz, and the videos were analyzed with NIS Elements software.

### LiquidBots Capture of Microplastics

A magnetic setup was used for navigation and adsorption experiments with amino‐modified PS microplastics (Merck, 2 µm) under a 3 mT magnetic field at 1 Hz. Video recording was performed at 25 frames per second using a Nikon ECLIPSE TS2R inverted microscope and Pylon Viewer software.

### PEG Degradation

The light degradation capability of LiquidBots was assessed on PEG (Mw: 4000, Alfa Aesar) under light irradiation. A mixture of 1 mg PEG 4000 and 10 mg microrobots was added to 1 mL of deionized water containing 1 wt.% H_2_O_2_ in disposable cuvette. The cuvette was irradiated for 6 h using a 365 nm UV LED lamp (9 W) as the light source. A control sample was prepared without LiquidBots. The cuvette was exposed to natural light for 6 h, after which a magnet was used to separate the LiquidBots, and the remaining solution was collected for analysis. The samples were analyzed using an Ultraflextreme instrument (Bruker Daltonics, Bremen, Germany) in linear positive ion detection mode. A three‐layer preparation technique was employed: 0.2 µL of 50 mg mL^−1^ trans‐2‐[3‐(4‐tert‐butylphenyl)‐2‐methyl‐2‐propenylidene] malononitrile in acetone was dried on a stainless‐steel target, followed by 0.2 µL of saturated NaI solution and then 0.5 µL of the sample solution.

Regenerablility of LiquidBots: LiquidBots were extracted from the water using an external magnet and then ultrasonicated in 5 m HCl for 2 min to remove surface Ga_2_O_3_ and re‐coalesce the GaIn‐Fe structure.

## Conflict of Interest

The authors declare no conflict of interest.

## Author Contributions

X.W. performed the fabrication of the LiquidBots, recorded and analyzed their motion, conducted the capture of microplastics and degradation of PEG, and wrote the manuscript. X.P. performed SEM‐EDX analysis and XRD and VSM measurements. Xiaohui Ju performed XPS measurement and analysis. X.W. designed the experiments. X.W. originated the idea. M.P. provided the research direction and funding. M.P., J.G., and L.R supervised the project. All authors contributed to writing the manuscript.

## Supporting information



Supporting Information

Supplemental Movie 1

Supplemental Movie 2

## Data Availability

The data that support the findings of this study are available from the corresponding author upon reasonable request.
