# Supplementary material for: Reconfigurable Self‐Assembling Photocatalytic Magnetic Liquid Metal Microrobot Swarm for Microplastic Capture and Degradation
Source: Small. 2025 Aug 27;21(38):2501351. doi: 10.1002/smll.202501351 (PMC12462602; doi:10.1002/smll.202501351)
Supplement: Supplementary file 1 — Supporting Information [file SMLL-21-2501351-s002.docx]

**Reconfigurable Self-assembling Photocatalytic Magnetic Liquid Metal Microrobot Swarm for Microplastic Capture and Degradation**

Xianghua Wu,^1,2^ Xia Peng,^1^ Long Ren,^2^ Jianguo Guan,^2,3^ Martin Pumera^1,4,5^*

^1^Future Energy and Innovation Laboratory, Central European Institute of Technology, Brno University of Technology, Purkynova 123, Brno, 61200, Czech Republic

^2^State Key Laboratory of Advanced Technology for Materials Synthesis and Processing, International School of Materials Science and Engineering, Wuhan University of Technology, 122 Luoshi Road, Wuhan, 430070, China

^3^Wuhan Institute of Photochemistry and Technology, 7 North Bingang Road, Wuhan, 430083, China

^4^Department of Medical Research, China Medical University Hospital, China Medical University, No. 91 Hsueh-Shih Road, Taichung, TW-40402, Taiwan

^5^Advanced Nanorobots & Multiscale Robotics Laboratory, Faculty of Electrical Engineering and Computer Science, VSB -Technical University of Ostrava, 17. listopadu 2172/15, Ostrava, 70800, Czech Republic

* Author for correspondence: martin.pumera@ceitec.vutbr.cz


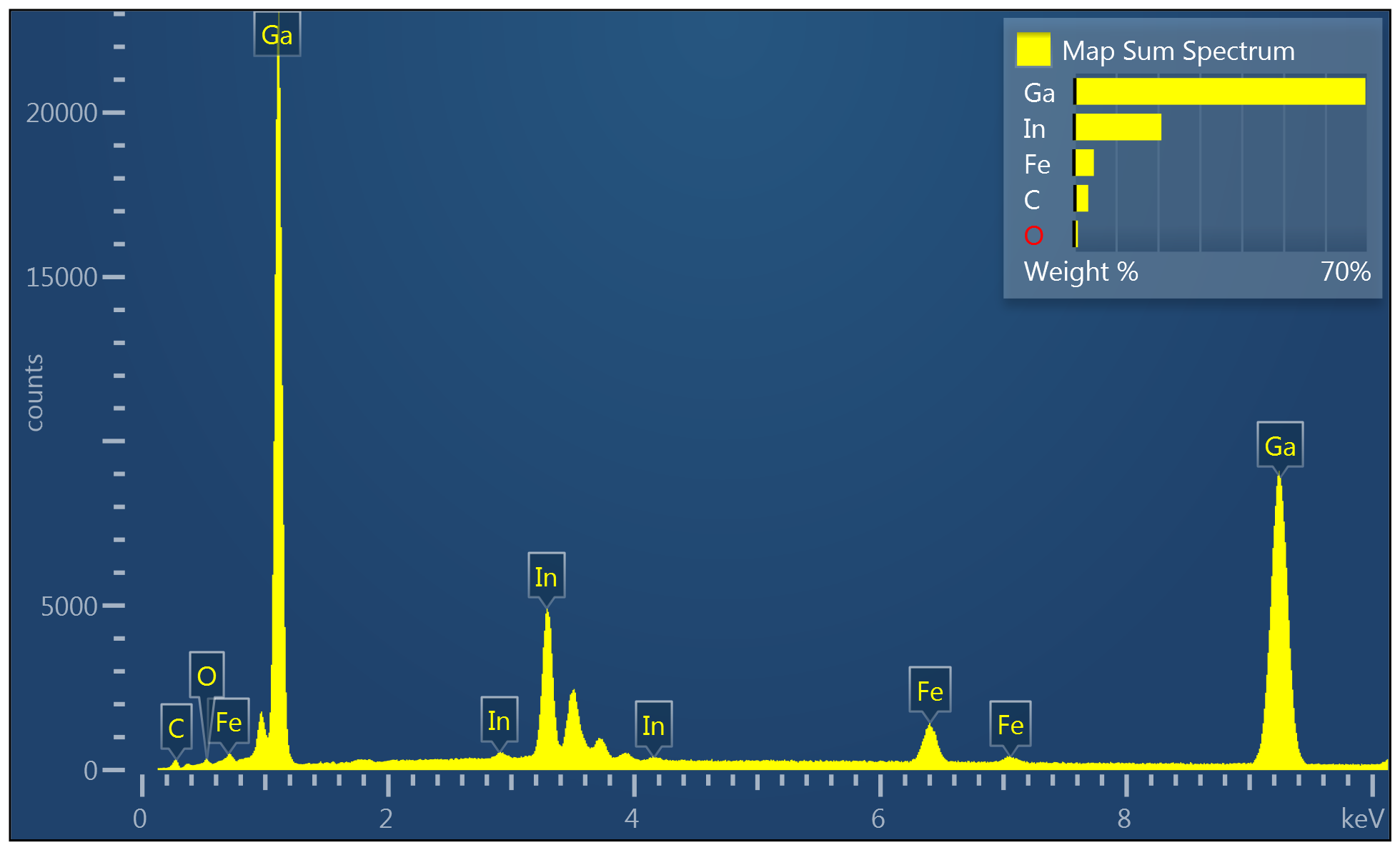


Figure S1. EDX spectrum of the LiquidBots.


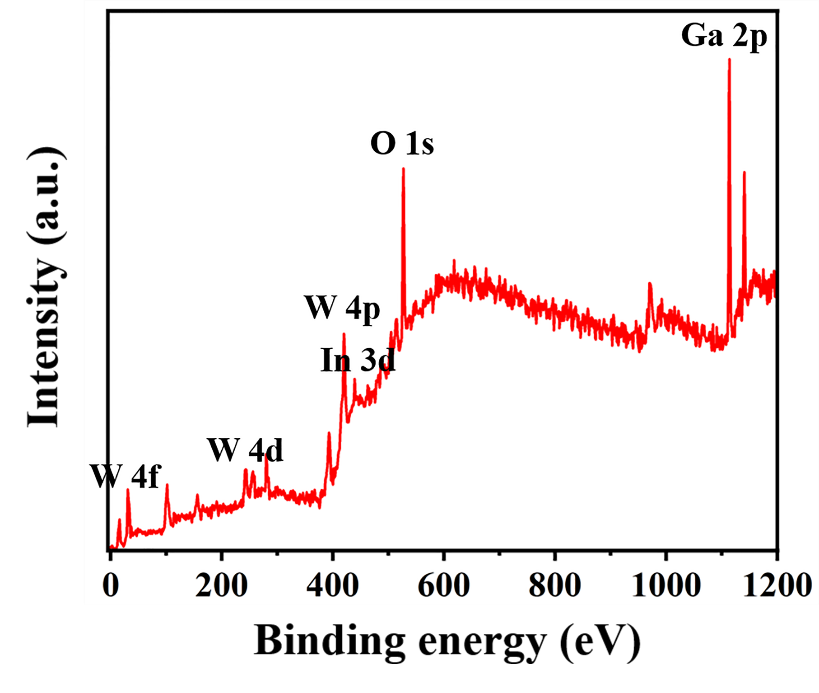


Figure S2. XPS of LiquidBots.


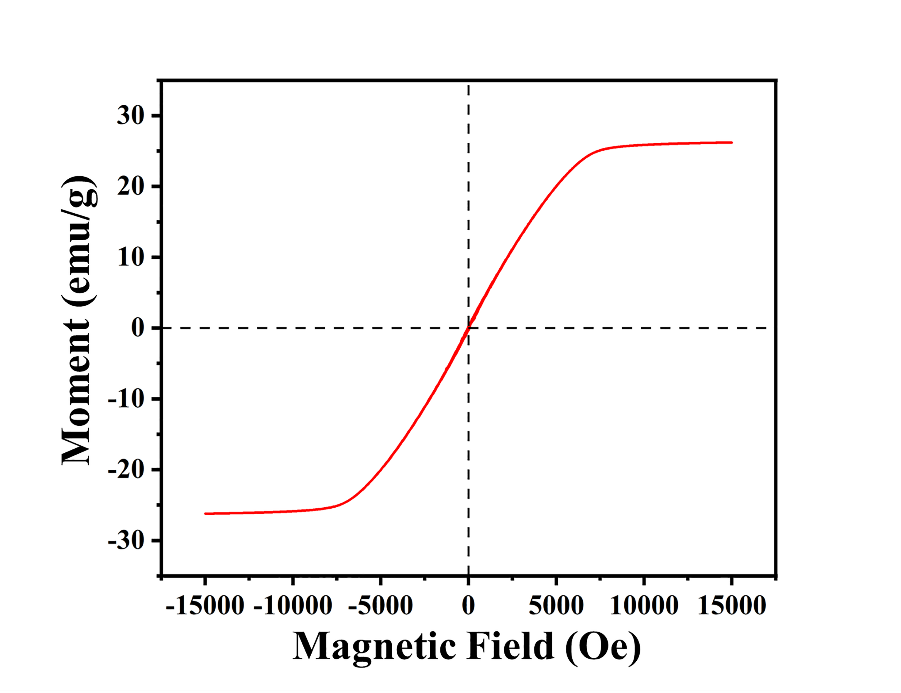


Figure S3. VSM of LiquidBots.


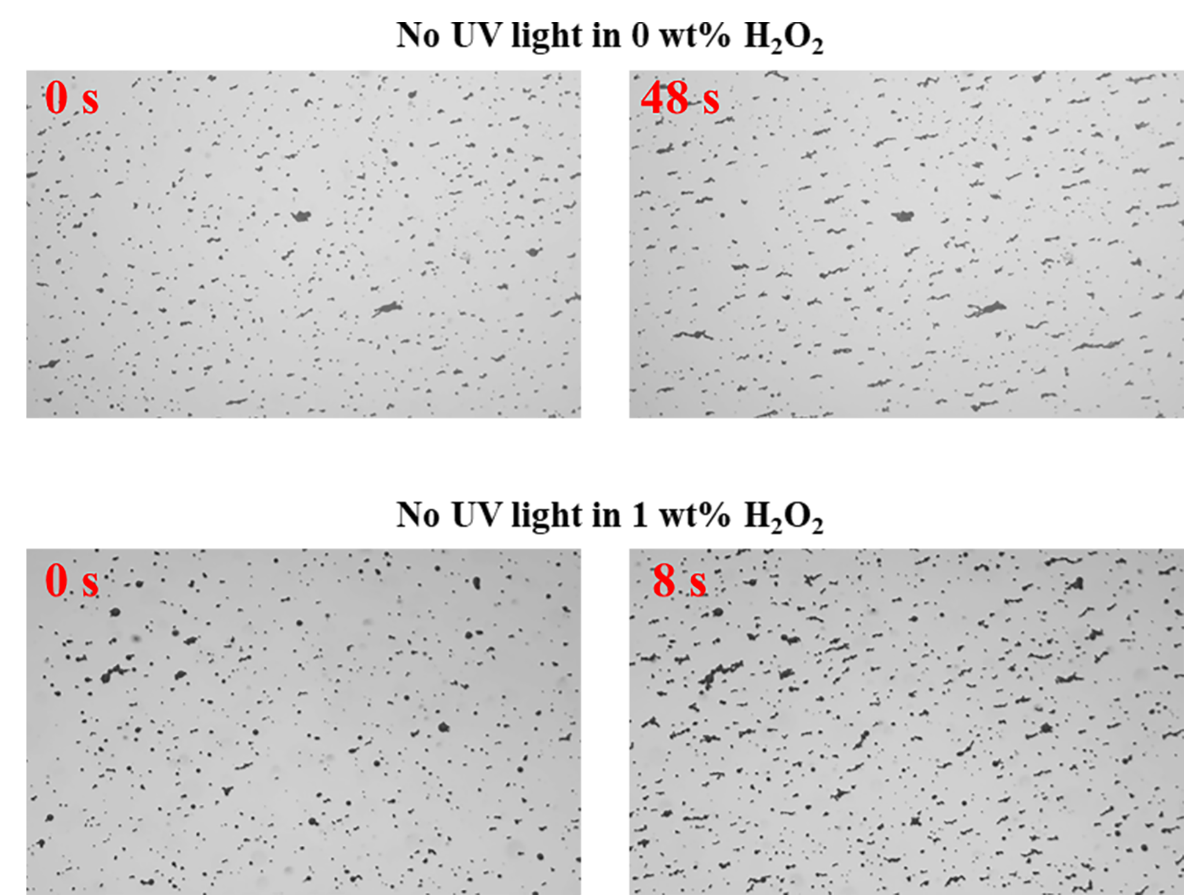


Figure S4. Self-assembly of LiquidBots in different concentrations of H_2_O_2_ without UV-light irradiation.


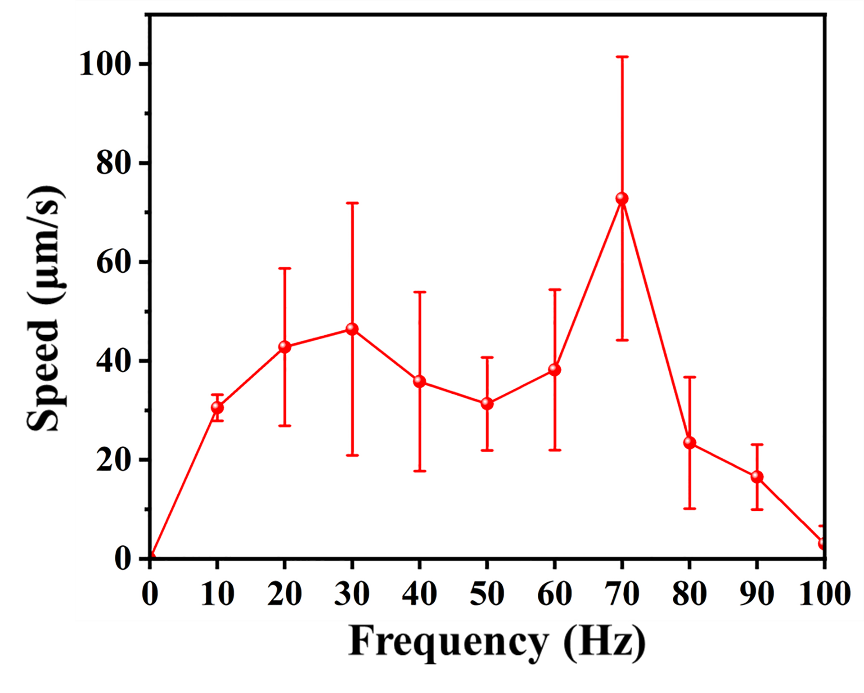


Figure S5. Speed of the LiquidBots under different magnetic field frequencies in rolling mode. Error bars represent the standard deviation; n = 10 independent replicates.


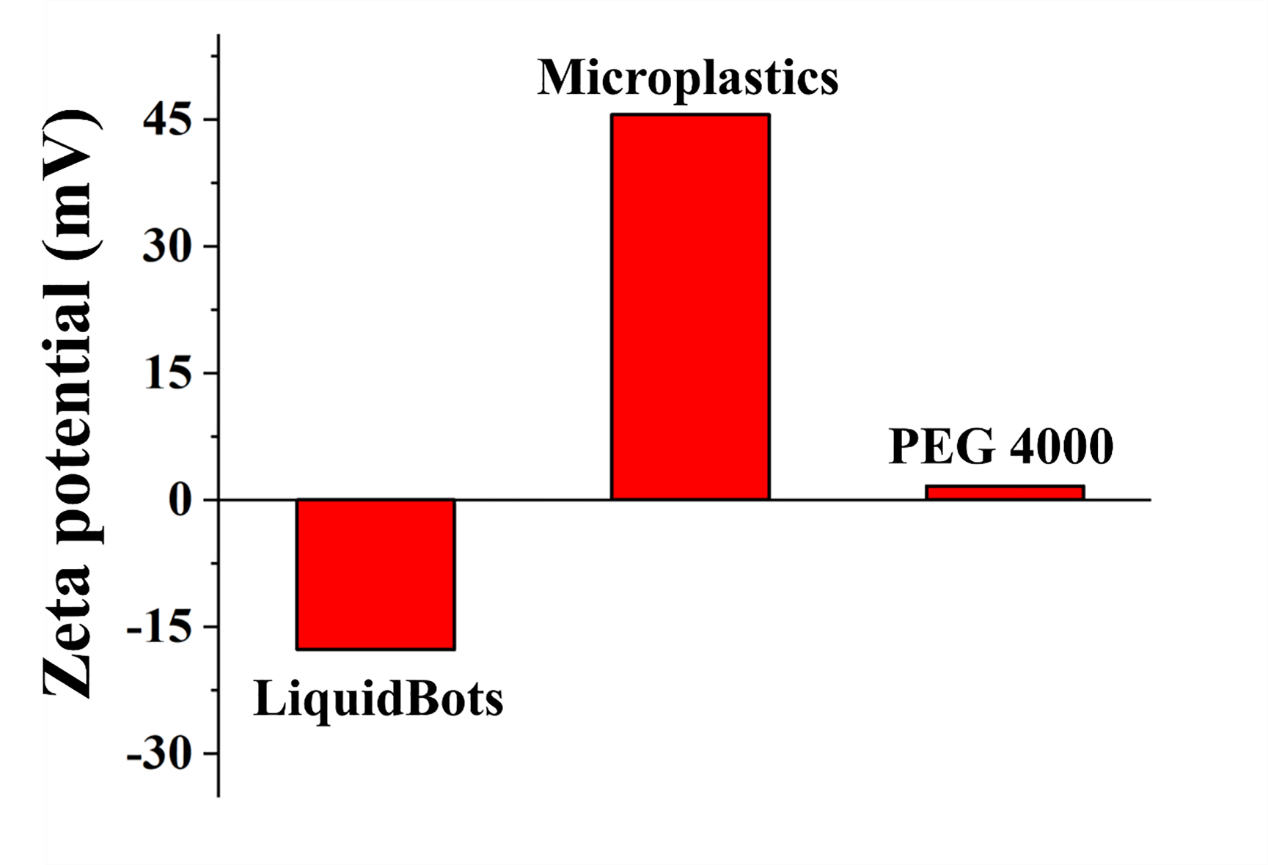


Figure S6. Zeta potential of LiquidBots, microplastics, and PEG 4000.


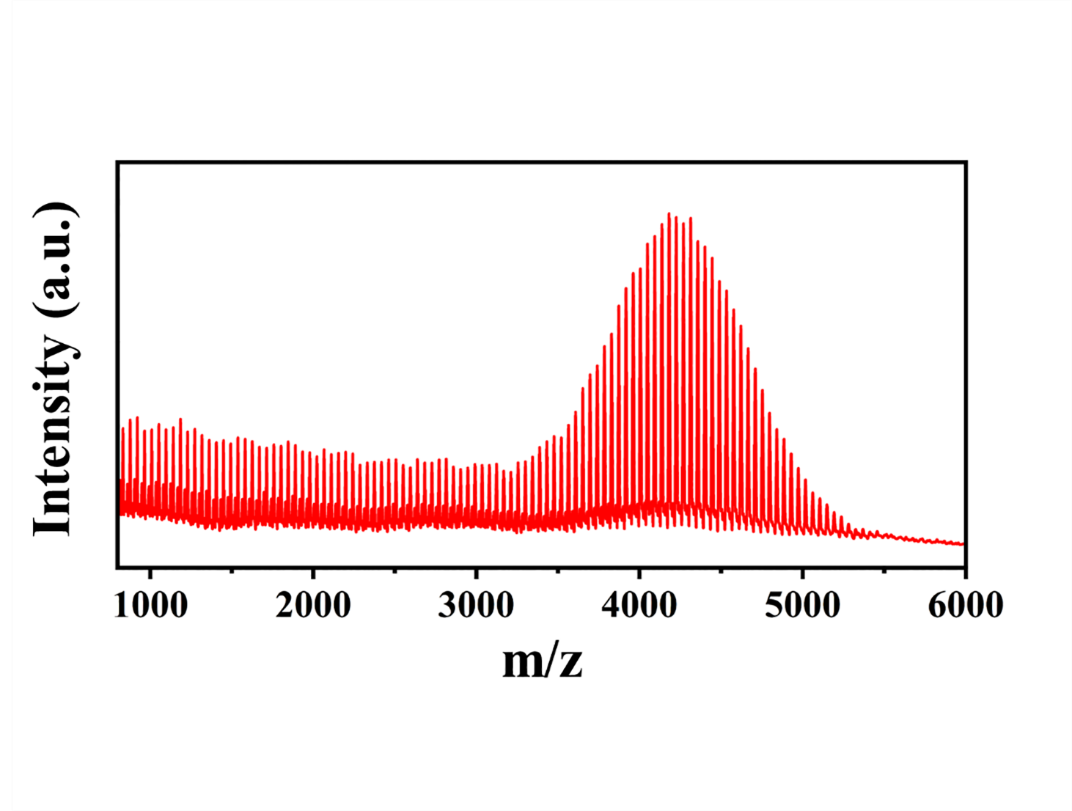


Figure S7. MALDI-MS spectrum of PEG after 6 hours of treatment with 1 wt% H₂O₂.


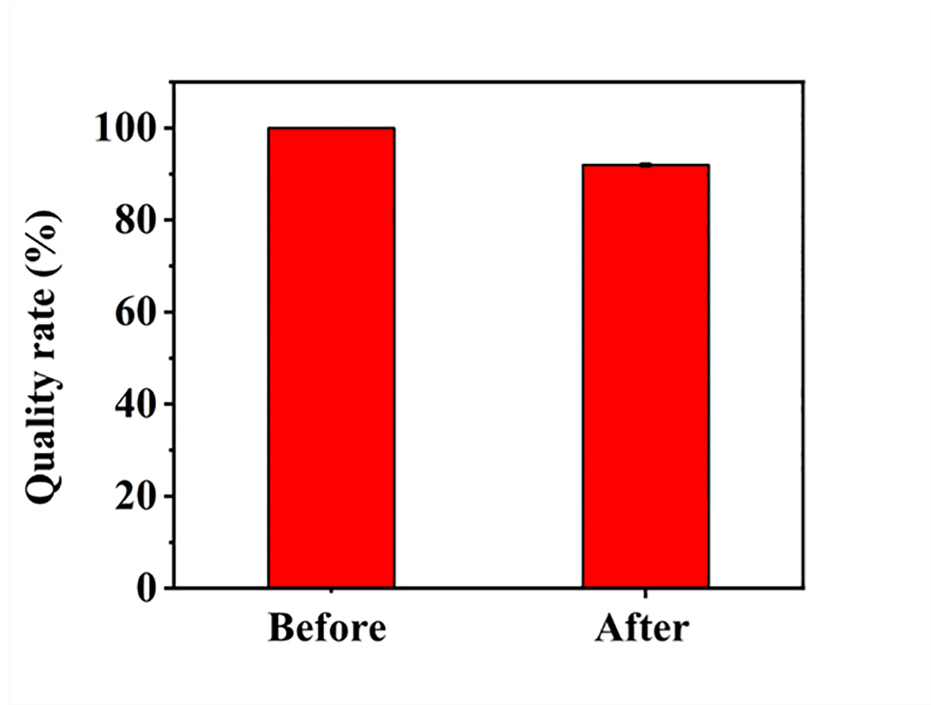


Figure S8. Quantitative comparison before and after LiquidBots re-coalesce into GaIn-Fe. Error bars represent the standard deviation; n = 3 independent replicates.
